# ZFP36 disruption is insufficient to enhance the function of mesothelin-targeting human CAR-T cells

**DOI:** 10.1038/s41598-024-53769-8

**Published:** 2024-02-07

**Authors:** David Mai, Tifara Boyce, Aakash Mehta, Jordan Reff, John Scholler, Neil C. Sheppard, Carl H. June

**Affiliations:** 1 Department of Bioengineering, University of Pennsylvania, Philadelphia, PA USA; 2 Center for Cellular Immunotherapies, Perelman School of Medicine, Philadelphia, PA USA; 3 Department of Biology, University of Pennsylvania, Philadelphia, PA USA; 4 The Wharton School, University of Pennsylvania, Philadelphia, PA USA; 5 Department of Pathology and Lab Medicine, Perelman School of Medicine, Philadelphia, PA USA

**Keywords:** Cancer therapy, Immunotherapy

## Abstract

Loss of inflammatory effector function, such as cytokine production and proliferation, is a fundamental driver of failure in T cell therapies against solid tumors. Here, we used CRISPR/Cas9 to genetically disrupt ZFP36, an RNA binding protein that regulates the stability of mRNAs involved in T cell inflammatory function, such as the cytokines IL2 and IFNγ, in human T cells engineered with a clinical-stage mesothelin-targeting CAR to determine whether its disruption could enhance antitumor responses. ZFP36 disruption slightly increased antigen-independent activation and cytokine responses but did not enhance overall performance in vitro or in vivo in a xenograft tumor model with NSG mice. While ZFP36 disruption does not reduce the function of CAR-T cells, these results suggest that singular disruption of ZFP36 is not sufficient to improve their function and may benefit from a multiplexed approach.

## Introduction

Adoptive T cell therapies, particularly chimeric antigen receptor (CAR) T cell therapy, have been transformative for the treatment of hematological malignancies, with currently 6 FDA-approved CAR-T cell treatments for various CD19 and B cell maturation antigen targeted B cell malignancies^1–4^. However, there are still no effective T cell therapies against solid tumors due to various intrinsic and extrinsic factors that induce T cell hypofunction and loss of inflammatory effector function in those settings^5,6^. Recent work has highlighted the promise of disrupting the class of RNA binding proteins (RBPs) that regulate T cell inflammatory activity by binding to the 3’UTRs of transcripts encoding inflammatory factors such as cytokines, co-stimulatory receptors, and transcription factors^7–11^. These RBPs include Regnase-1, Roquin-1, and ZFP36 family proteins and have known roles for preventing autoreactive and hyperinflammatory T cell responses in mice and humans^12–16^. While Regnase-1 and Roquin-1 disruption have now been investigated in several models of adoptive T cell therapy, to our current knowledge, ZFP36 family proteins have been less explored^7–11^.

Here, we show that disruption of ZFP36 in primary human T cells bearing a clinical-stage mesothelin-targeting CAR (mesoCAR) is not sufficient to increase therapeutic function. While knockout of ZFP36 somewhat increased antigen-independent activation in CD4 CAR-T cells and cytokine secretion, ZFP36-KO cells performed similarly to unedited mock cells in a xenograft tumor model and exhibited similar cytotoxicity, proliferation, and cytokine responses in antigen settings. These findings suggest a greater redundancy in the role of ZFP36 proteins, perhaps compared to Regnase-1 or Roquin-1, and that multiplex disruption of ZFP36 proteins may be needed to enhance the inflammatory function of engineered T cells in therapeutic contexts.

## Results

### ZFP36 family proteins are transcriptionally highly expressed compared to regulatory RBPs Regnase-1 and Roquin-1

Regulatory RBPs, such as Regnase-1 and Roquin-1, are crucial in their roles to restrict aberrant inflammation in immune cells, including T cells^12–14^. In the context of cancer, however, it is beneficial for T cells to maintain or increase the potency of their inflammatory function. Recent work has suggested that perturbation of regulatory RBPs Regnase-1 and Roquin-1 can increase the antitumor function of adoptive T cells therapies^7–11^. As a result, we hypothesized that disruption of other regulatory immune RBPs could produce a similar effect in therapeutic T cells.

**Figure 1:**
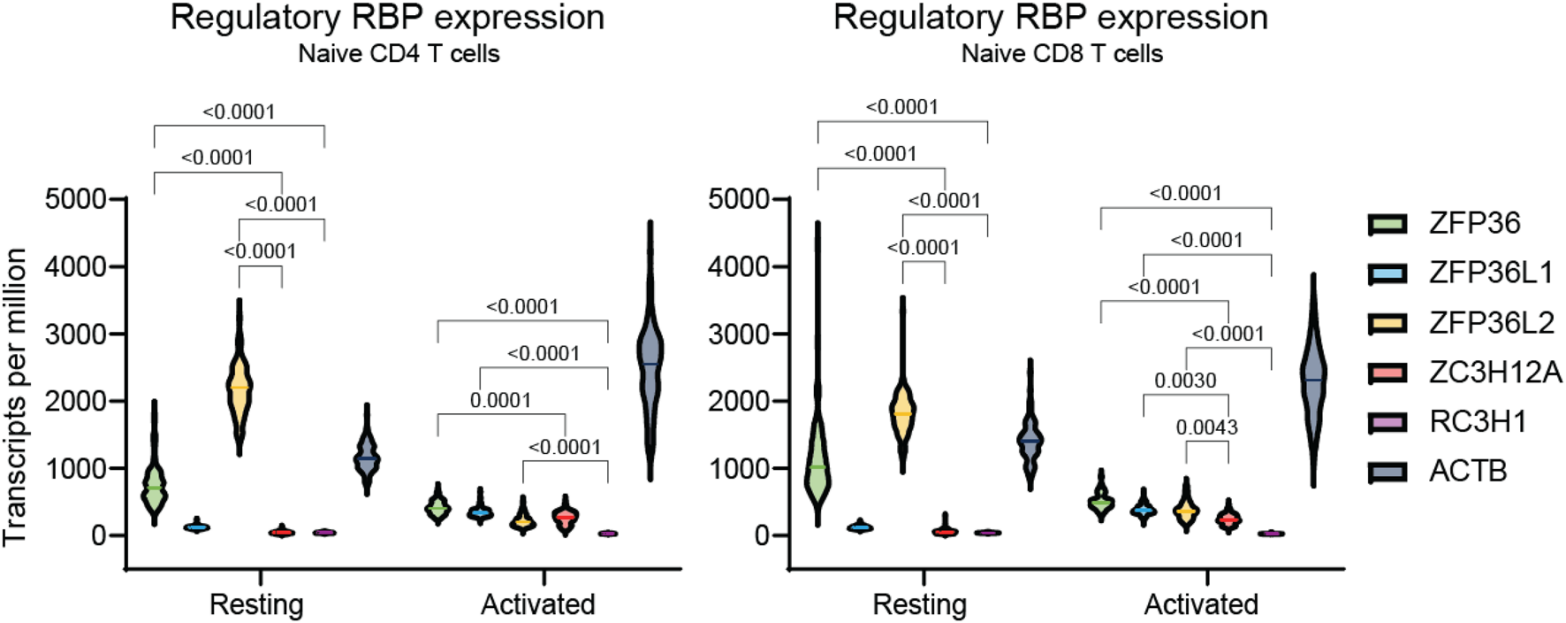
Transcriptional expression levels of regulatory immune RBPs in naïve human T cells. Transcript levels of the ZFP36 family of RBPs, Regnase-1 (ZC3H12A), Roquin-1 (RC3H1), and β-actin (ACTB) as a housekeeping gene control in resting and activated CD4 (left) and CD8 (right) human T cells. Expression data depicted is derived from bulk RNA sequencing experiments performed on multiple healthy human donors (n > 3) and taken from the Database of Immune Cell Expression, Expression quantitative trait loci and Epigenomics (DICE). Line represents median. Two-way ANOVA followed by Tukey’s multiple comparisons tests was used for statistical analysis.

ZFP36 family proteins are known regulators of T cell activation and can restrain immune responses^15–17^. Their disruption in mice has been shown to accelerate and enhance antiviral immunity as well as allow T cells to increase the production of proinflammatory cytokines such as IFNγ, TNFα, and GM-CSF^15–17^. To gain insight into the relative transcriptional activities of these RBPs in T cells we compared transcriptional data of the ZFP36 family proteins to Regnase-1 and Roquin-1 using publicly available transcriptional data taken from bulk RNA sequencing experiments performed on healthy human donors curated through the Database of Immune Cell Expression, Expression quantitative trait loci and Epigenomics (DICE)^18^. In a resting state, ZFP36 and ZFP36L2 were the most highly expressed, significantly greater than Regnase-1 and Roquin-1 transcriptional expression across both naïve CD4 and CD8 T cells suggesting these two ZFP36 family proteins as potentially interesting targets for genetic ablation (Fig. 1). Given the limited work on the disruption of these proteins in a T cell therapy context and the roles of ZFP36L1 and ZFP36L2 on antigen-specific clonal expansion, we focused efforts on ZFP36 whose function has been better characterized^16^.

### Genetic disruption of ZFP36 does not affect human CAR-T cell manufacturing or phenotypic profile

**Figure 2:**
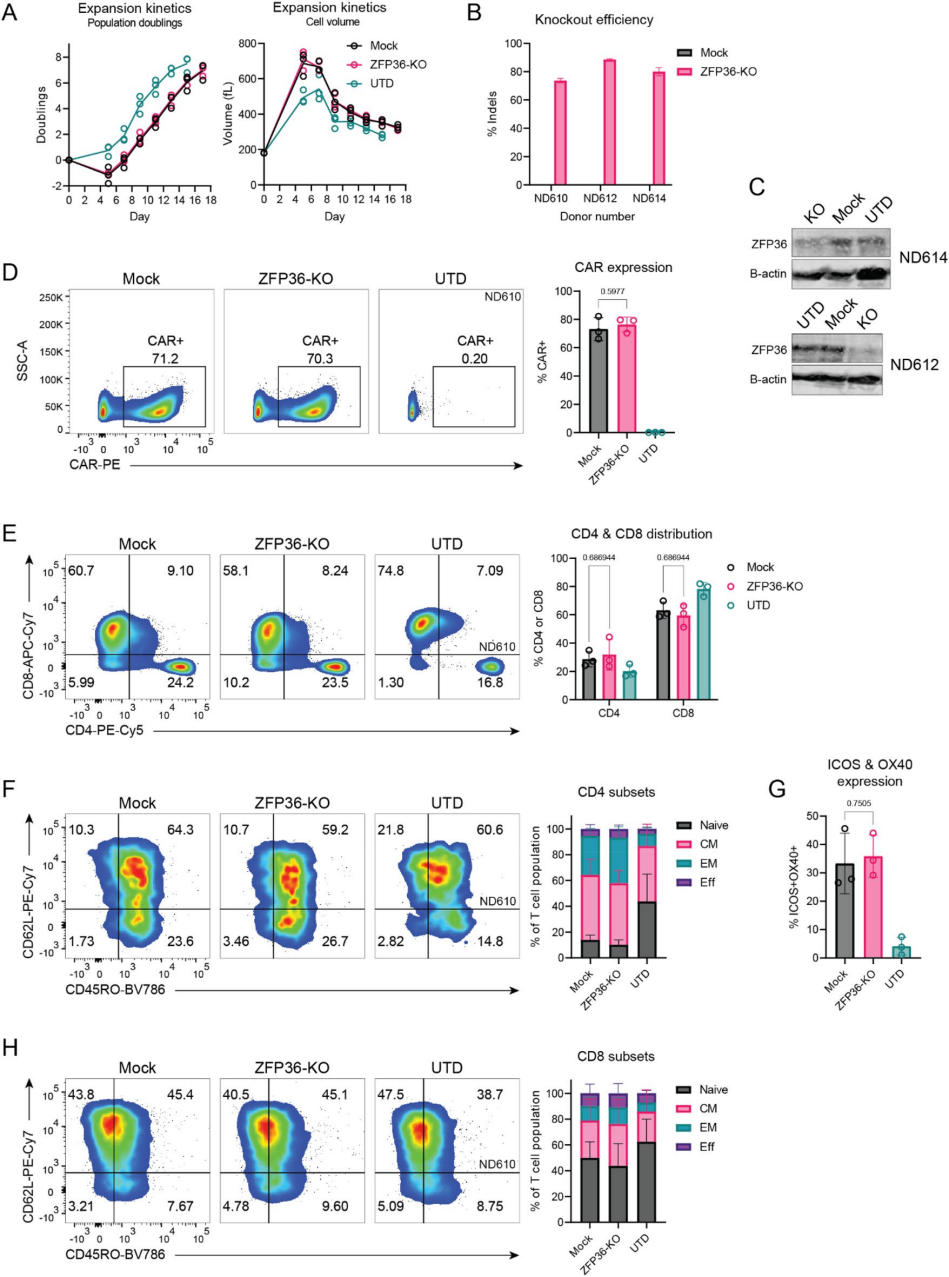
Manufacturing and baseline phenotype of ZFP36-KO mesoCAR-T cells. (**A**) Expansion kinetics of ZFP36-KO CAR-T cells (n = 3 donors). Population doublings (left) and cell volume (right) over time are shown. Hollow circles represent individual donors and solid lines represent the average of all donors. (**B**) Knockout efficiency of ZFP36 in CAR-T cells (n = 3 donors). Error bars represent SD. (**C**) Representative western blots demonstrating protein level disruption of ZFP36 with loading controls. (**D**) Representative flow plots (left) and summarized graph (right) showing CAR expression after CAR-T cells were thawed and rested overnight (n = 3 donors). Error bars represent SD. A two-tailed unpaired t-test was used for statistical analysis. (**E**) Representative flow plots (left) and summarized graph (right) showing CD4 and CD8 distribution after CAR-T cells were thawed and rested overnight (n = 3 donors). Error bars represent SD. Two-tailed unpaired t-tests were used for statistical analysis. (**F,H**) Representative flow plots (left) and summarized graph (right) of (**F**) CD4 and (**H**) CD8 T cell subsets by CD45RO and CD62L staining (n = 3 donors). Error bars represent SD. (**G**) Expression of co-stimulatory receptors ICOS and OX40 on resting CD4 T cells (n = 3 donors). Error bars represent SD. A two-tailed unpaired t-test was used for statistical analysis. UTD = untransduced T cells.

To test whether inhibition of ZFP36 would increase the functionality of human CAR-T cells in a solid tumor antigen context, we used CRISPR/Cas9 to genetically disrupt ZFP36 in human mesoCAR-T cells. We used a standard preclinical protocol to manufacture KO CAR-T cells as previously described^19^. Single guide RNAs (sgRNAs) were screened against ZFP36 and a top sgRNA was used for subsequent experiments. Disruption of ZFP36 did not alter the number of population doublings in mesoCAR-T cells during manufacture nor did it change the rate at which they rested down as measured by T cell volume (Fig. 2A). We were able to achieve high knockout efficiency across three independent donors and confirmed protein level disruption by western blot (Fig. 2B, C and Supplementary Fig. 1). After manufacture, cryopreservation, and subsequent thawing, we compared the baseline profiles of KO and mock mesoCAR-T cells, measuring CAR expression, CD4 and CD8 distribution, and T cell subpopulations within CD4 and CD8 subsets. Both CAR expression and CD4 and CD8 distributions were unaffected by ZFP36 disruption (Fig. 2D, E). Among both CD4 and CD8 T cell subsets, we also did not observe significant changes in the distribution of memory and effector subpopulations (Fig. 2F, H). As other inflammatory regulators, such as Roquin-1, are known to regulate costimulatory receptors ICOS and OX40, we also looked at the percentage of ICOS and OX40 double positive CD4 T cells, but this analysis also did not yield significant differences (Fig. 2G)^20,21^.

**Figure 3:**
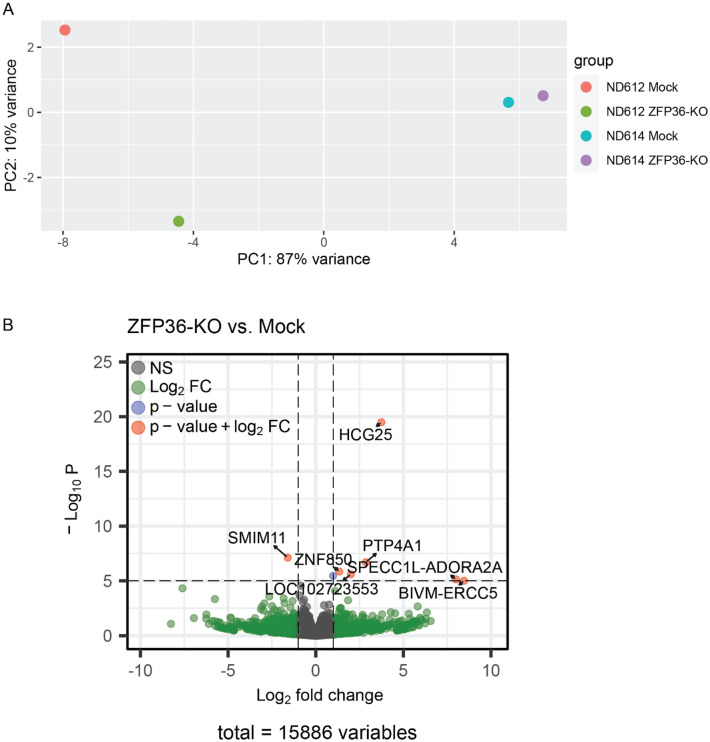
Bulk RNAseq of resting ZFP36-KO mesoCAR-T cells. (**A**) PCA clustering of ZFP36-KO and mock CAR-T cells (n = 2 donors). (**B**) Volcano plot showing differentially expressed transcripts between ZFP36-KO and mock CAR-T cells (n = 2 donors). Genes that are statistically significant (plotted p ≤ 10^–5^) and have a Log2FC ≥ 1 are shown in red. Statistical significance was calculated using the Wald test with Benjamini–Hochberg multiple testing correction.

ZFP36 is well established as an RBP that acts on AREs located at the 3′ UTRs of mRNAs^22–25^. As a result, we also sought to profile the transcriptome of KO CAR-T cells to determine whether ZFP36 disruption leads to an altered transcriptional resting state. Using bulk RNA sequencing, we quantified the transcriptional profiles of resting CAR-T cells in two independent donors. PCA clustering did not reveal a notable pattern between KO and mock and showed that most of the variance among samples was due to biological differences between the donors, a well-known source of variation (Fig. 3A). Differential expression analysis between KO and mock groups also demonstrated that ZFP36 disruption does not significantly impact the resting transcriptomic state of mesoCAR-T cells, as only a handful of genes were differentially expressed, and none that were notably related to inflammatory processes (Fig. 3B). One consideration may be that while other inflammatory regulators such as Regnase-1 or Roquin-1 actively suppress the expression of inflammatory transcripts during resting states, ZFP36 family proteins have been reported to be more important in shaping early activation responses after stimulation^15,23^. In addition, the functional redundancy of ZFP36 family proteins in T cells may be greater than that of Roquin (Roquin-1, Roquin-2) and Regnase (Regnase-1, Regnase-2, Regnase-3, Regnase-4) family proteins, which may obscure transcriptional differences between resting mock and ZFP36-KO T cells^16^.

### ZFP36-KO CAR-T cells retain similar activation function

**Figure 4:**
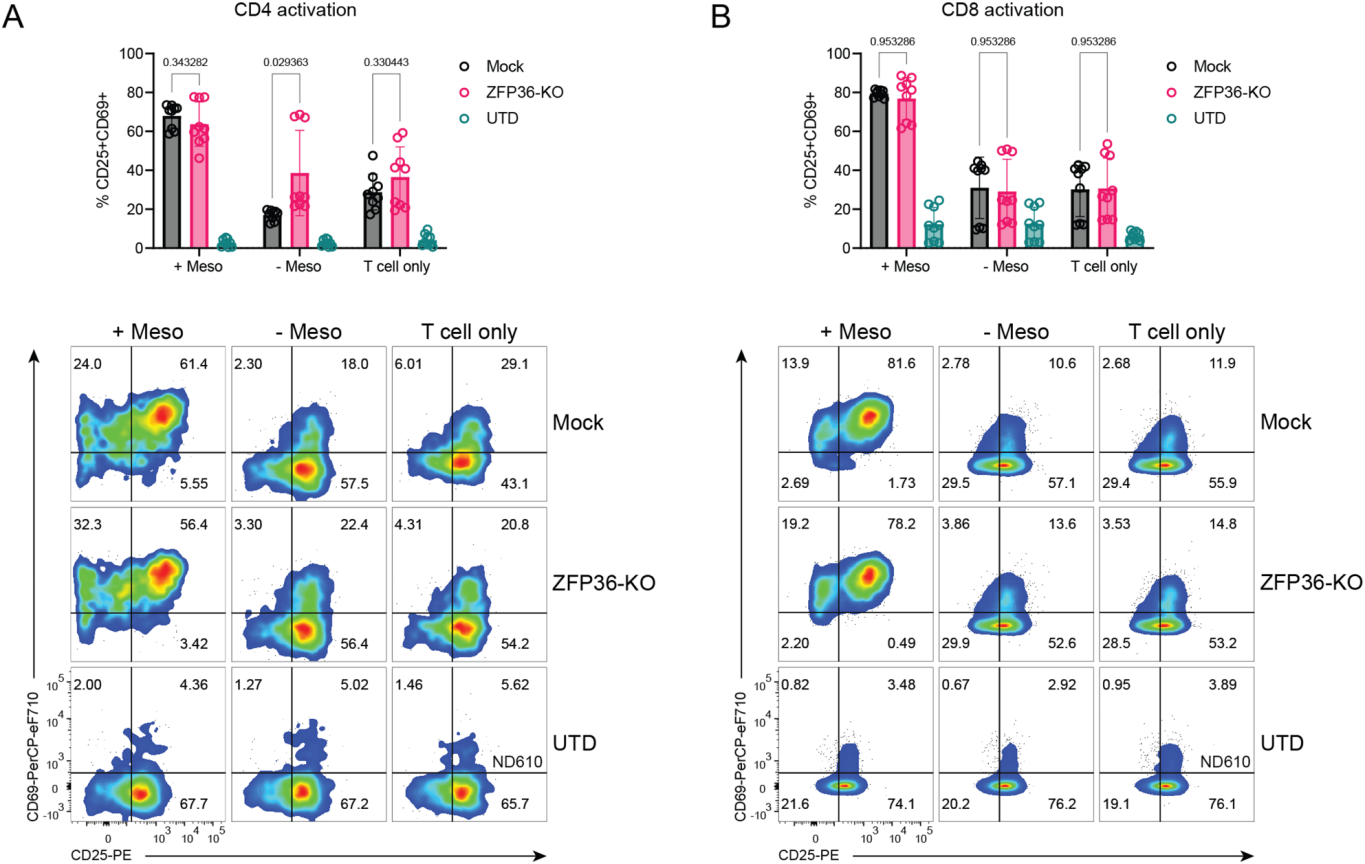
Activation profile of ZFP36-KO mesoCAR-T cells. (**A,B**) Activation of (**A**) CD4 and (**B**) CD8 ZFP36-KO and mock CAR-T cells with and without antigen stimulation as determined by CD25 and CD69 expression (n = 3 donors). Summarized CD25 and CD69 double positive T cells (top) and representative flow plots (bottom) from 3 donors and 3 experiments performed in triplicate. Error bars represent SD. Multiple unpaired t-tests were used for statistical analysis. UTD = untransduced T cells.

Because ZFP36 family proteins are known to coordinate early activation processes after initial stimulation, we sought to characterize the effects of ZFP36 disruption on CD25 and CD69 expression in CAR-T cells with and without antigen stimulation^15,17^. ZFP36 KO did not alter antigen-induced CD25 and CD69 expression in CD4 or CD8 mesoCAR-T cells, nor did it change the percentage of CD25+ CD69+ cells without tumor cells (Fig. 4A, B). Disruption of ZFP36 in CD4 mesoCAR-T cells cocultured with antigen-negative tumor cells led to a statistically significant, slight increase in CD25+ CD69+ population; however, this difference was notable only in one of three donors and is not a strong phenotypic difference (Fig. 4A). This suggests that ZFP36 may have a subtle role in the activation potential of engineered CD4 T cells, which may be explored in future work.

### ZFP36-KO CAR-T cells function comparably to unedited CAR-T cells

**Figure 5:**
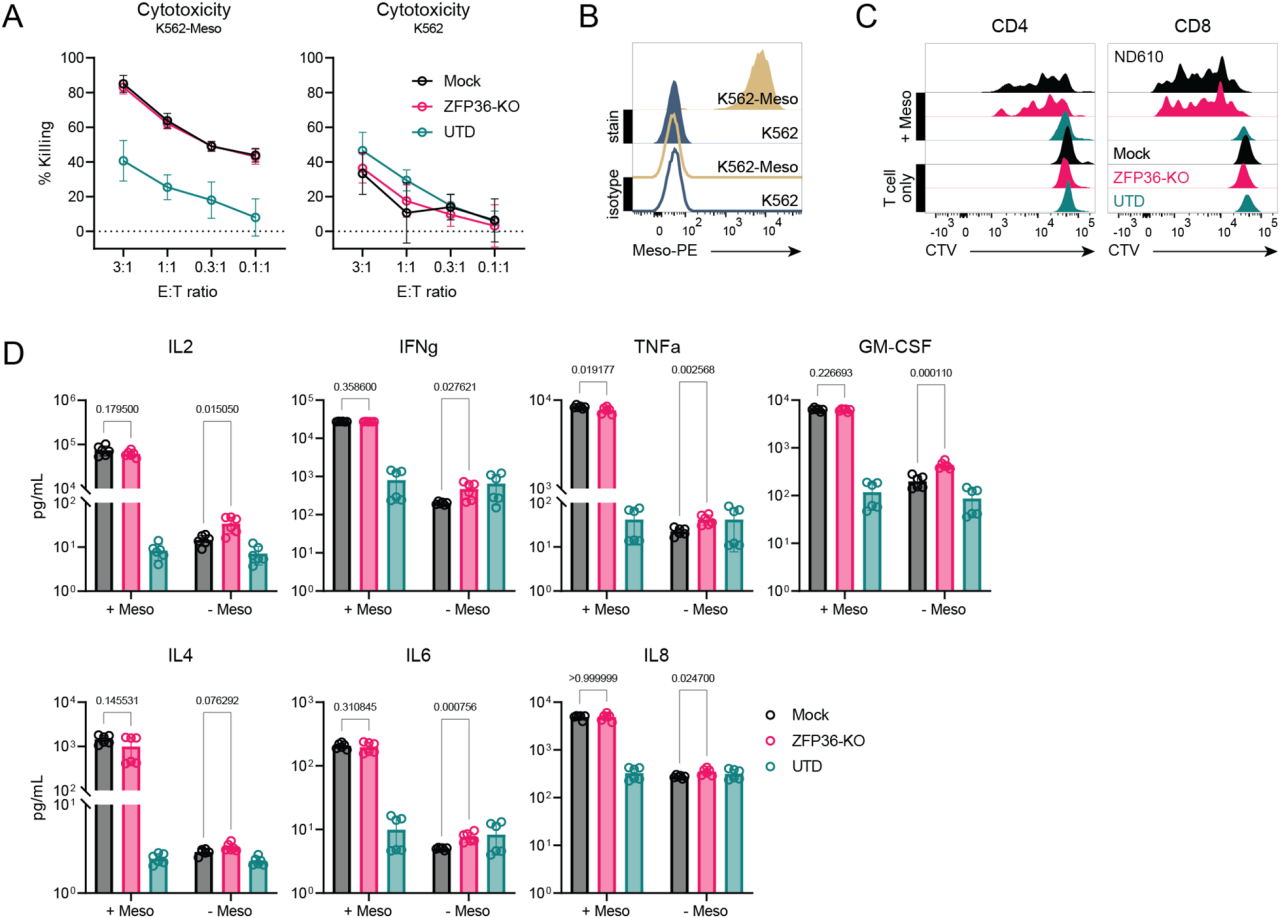
Functional in vitro profiling of ZFP36-KO mesoCAR-T cells. (**A**) Cytotoxicity of CAR-T cells against (left) antigen-positive and (right) -negative cells over a range of effector to target ratios. Data shown is pooled from 3 donors and 3 experiments performed in triplicate. Error bars represent SD. (**B**) Mesothelin antigen expression on cell lines used for in vitro assays. (**C**) Representative flow plots showing proliferation of (left) CD4 and (right) CD8 CAR-T cells with and without stimulation from antigen-positive cells (n = 3 donors). (**D**) Quantification of cytokine secretion against antigen-positive and antigen negative cells. Data shown is pooled from 2 donors and 2 experiments performed in triplicate. Error bars represent SD. Multiple unpaired t-tests were used for statistical analysis. UTD = untransduced T cells.

We additionally characterized the in vitro and in vivo function of KO and mock mesoCAR-T cells. ZFP36 KO did not alter cytotoxic function of mesoCAR-T cells in both antigen-positive and antigen-negative contexts (Fig. 5A, B). Proliferation in the presence and absence of antigen were also unaffected by ZFP36 disruption (Fig. 5C). While ZFP36 has been shown to regulate proinflammatory transcripts including those encoding cytokines, we did not observe significant differences between effector cytokine secretion when CAR-T cells were cultured with mesothelin-expressing K562 cells (Fig. 5D). However, similar to the observed increase in CD25 and CD69 expression in KO CD4 mesoCAR-T cells, we found a slight antigen-independent increase in cytokine secretion for IL2, IL6, IFNγ, TNFα, GM-CSF and IL8, however the fold-change was < 2-fold in each case and thus unlikely to have functional impact (Fig. 5D). These results suggest that while disruption of ZFP36 alone may have subtle phenotypic effects in unstimulated mesoCAR-T cells, these effects are not large enough to be consequential or are not present in an activated context.

**Figure 6:**
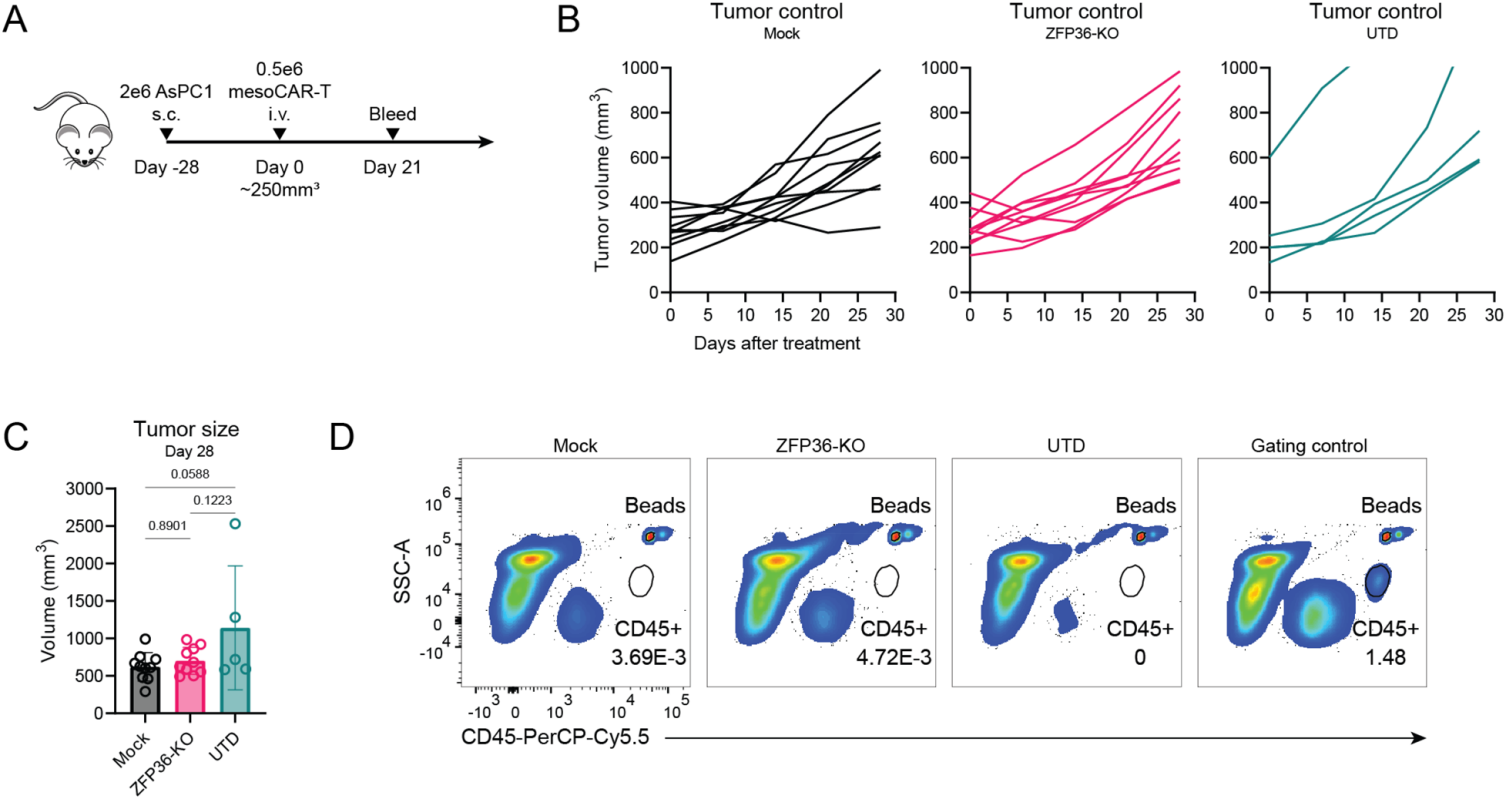
In vivo performance of ZFP36-KO mesoCAR-T cells at a subtherapeutic dose. (**A**) Experimental scheme for profiling in vivo function of CAR-T cells at a subtherapeutic dose in NSG mice. (**B**) Tumor growth over time for mice treated with CAR-T cells or UTD control. (**C**) Tumor sizes at 28 days after CAR-T cell treatment. Data shown for (**B,C**) is pooled from 2 donors and 2 experiments. Error bars represent SD. One-way ANOVA followed by Tukey’s multiple comparisons tests was used for statistical analysis. (**D**) Representative flow plots depicting lack of CD45+ cells in the peripheral blood at 21 days after CAR-T cell treatment with a gating control from a higher dose treatment. UTD = untransduced T cells.

To interrogate the function of KO mesoCAR-T cells in vivo, we leveraged a xenograft tumor model in NSG mice used previously in our lab (Fig. 6A). NSG mice were subcutaneously engrafted with mesothelin positive AsPC1 pancreatic cancer tumor cells to establish large tumors averaging 250 mm^3^ and treated under stress-test conditions with a low dose of 0.5 × 10^6^ CAR-T cells. Previous work using this low dose regimen to treat tumors at this size demonstrated it was subtherapeutic for mock mesoCAR-T cells and was able to discern differences between KO and unedited cells, even when no strong functional differences were observed for antigen-activated CAR-T cells in short-term in vitro assays^11^. Disruption of ZFP36 did not improve tumor burden control in this model (Fig. 6B, C). Enumeration of T cells in the blood supported these observations, which showed a relative absence of T cells at this dose (Fig. 6D). Taken together, these results suggest that while singular disruption of ZFP36 may slightly increase functional activity in unstimulated cells, it is not sufficient to increase the antitumor function of mesoCAR-T cells.

## Discussion

Here, we explore the effects of disrupting ZFP36 in human mesoCAR-T cells to determine whether loss of this regulatory RBP could enhance CAR-T cell inflammatory function in a solid tumor context. While previous work that focused on other regulatory RBPs such as Regnase-1 and Roquin-1 demonstrated notable increases in antigen-dependent inflammatory function upon disruption of these RBPs in adoptive T cell models, loss of ZFP36 did not enhance engineered T cell function, though it also did not diminish it. This may highlight differences in the regulatory roles of these RBPs, even though they serve similar purposes in restraining inflammatory responses. Additional considerations include the functional roles of these RBP paralogues as well as cooperativity between RBPs. For example, while Regnase-1 and Roquin-1 have distinct functions and targets, they have been shown to interact to regulate shared transcript targets^10,26^. As a result, disruption of either one RBP may abolish cooperative regulatory activity in addition to eliminating its individual function, producing a greater perceived effect of the single knockout. While there has been less work on these RBP paralogues, their roles may also influence the phenotypes of these knockouts. There are four paralogues of Regnase, which have been reported to function more prominently in specific cell types or contexts^27,28^. As a result, several studies have found that disruption of Regnase-1 alone in T cells is sufficient to increase inflammatory T cell function^7,9–11^. Meanwhile, ZFP36 family proteins together may have a more redundant and prominent role in T cells, leading to minimal phenotypic perturbations when only one ZFP36 family protein is disrupted. While this could be theoretically mitigated by multiplex disruption of two or all three ZFP36 family proteins, the nuances between hyperinflammatory antitumor activity and autoimmune pathology remain poorly defined. Furthermore, some of these proteins are important in the development of normal functioning T cells, though they may be less important in post-thymic T cells^16^. Future work investigating perturbation of multiple ZFP36 family proteins or together with other regulatory RBPs may elucidate potential benefits or consequences of this therapeutic strategy.

## Methods

### Primary T cells and tumor cell lines

Healthy donor PBMCs were obtained after written informed consent under a University Institutional Review Board-approved protocol and processed to isolate T cells by the Human Immunology Core (HIC) at the Perelman School of Medicine. All methods were carried out in accordance with University of Pennsylvania Institutional Review Board approval. All experimental protocols were carried out in accordance with University of Pennsylvania Institutional Review Board approval. Cell lines were obtained from the ATCC, ID verified by ATCC human cell line STR profiling, and confirmed negative for mycoplasma using Cambrex MycoAlert mycoplasma detection assay (Promega). AsPC-1 cells were grown in DF20 media consisting of DMEM/F12 (1:1) (Gibco, Life Technologies), 20% fetal bovine serum (Seradigm), and 1% penicillin/streptomycin (Gibco, Life Technologies). K562 and HEK293T cells were cultured in R10 media consisting of RPMI-1640 (Gibco, Life Technologies) with 10% FBS, 10 mM HEPES (Gibco), 1% GlutaMAX™ (Gibco), 1 mM Sodium Pyruvate (Gibco), 1% MEM NEAA (Gibco), and 1% penicillin/streptomycin. GFP/CBG-expressing and mesothelin-expressing cell lines were generated by lentiviral transduction for cell killing assays.

### Lentiviral vector production

Lentiviral vector production was performed as previously described^29^. Briefly, HEK293T cells were transfected with lentiviral mesoCAR and packaging plasmids using Lipofectamine 2000 (Invitrogen) following the manufacturer’s protocol. Lentiviral supernatants were collected at 24- and 48-h after transfection and concentrated using high-speed ultracentrifugation. To generate lentiviral stocks, the resulting concentrated lentivirus batches were resuspended in cold R10 media and stored in −80 °C.

### CAR-T cell production

The anti-mesothelin M5 4-1BB-zeta CAR is described in a clinical investigation (NCT03054298). CD4 and CD8 T cells were combined at a 1:1 ratio and rested overnight with R10 media supplemented with 5 ng/mL human IL7 and 5 ng/mL human IL15 (Preprotech). The next day, CRISPR-Cas9 editing was performed, and cells were cultured in cytokine-supplemented R10 media at 5 × 10^6^ cells/mL at 37C and activated 4–6 h later with Dynabeads^®^ CD3/CD28 CTS™ (Thermofisher) at a 3:1 bead-to-cell ratio at 1 × 10^6^ cells/mL. After 24 h, T cells were transduced at a multiplicity of infection of 3. At day 5, beads were removed from cultures. T cell cultures were maintained at 6 × 10^5^ cells/mL. Cell number and volume were monitored daily using Multisizer 3 Coulter Counter (Beckman). Transduced T cells were cryopreserved when they reached a rested state, as determined by cell size of around 300 fL.

### CRISPR-Cas9 editing

Single guide RNA (sgRNA) sequences targeting the ZFP36 genomic locus were designed using Benchling online software (https://www.benchling.com) and cross-validated with Synthego’s guide design verification tool (https://design.synthego.com/#/validate). sgRNA sequences were synthesized by Integrated DNA Technologies (IDT). After screening three sgRNAs against ZFP36, the top sgRNA was selected for downstream experiments (sgRNA sequence: 5′-CGCTCCACCAGCCTAGTGGA-3′). Gene disruption was performed following an optimized protocol previously described^19^. Briefly, T cells were washed three times in Opti-MEM™ reduced serum medium (Gibco) and resuspended at 1 × 10^8^ cells/mL in P3 nucleofection solution (Lonza). Ribonucleoprotein (RNP) complexes were generated by incubating each sgRNA (5ug per 10 × 10^6^ cells) individually with Cas9 nuclease (Aldevron, 10ug per 10 × 10^6^ cells) for 10 min at room temperature. For mock groups, no sgRNA was used and for multiple knockout groups, RNPs targeting each gene were incubated separately before combining. Cells were electroporated in batches of 10 × 10^6^ cells (100 µL) with a mixture of RNP complex and 16.8 pmol of electroporation enhancer (IDT) in electroporation cuvettes (electroporation code EH111) in a 4D-Nucleofector X-Unit (Lonza).

### Sequencing

Sanger sequencing of PCR-amplified genomic knockout regions was performed through the DNA Sequencing Facility at the University of Pennsylvania. PCR and sequencing primers are listed below:

PCR (5′ → 3′):

Forward: CCTGGGTCCCTCGGGATAA

Reverse: AAGCTGATGCTCTGGCGAAG

Sequencing (5′ → 3′):

Forward: CTCTGGGTTCCTGGCATCCG

Reverse: CCCTGGAGGTAGAACTTGTGAC

### Western blot

Western blotting was used to assess protein level quantification of ZFP36 in T cells. After primary expansion and prior to cryopreservation, 8 × 10^6^ T cells were pelleted and lysed in ice cold RIPA buffer (G Biosciences) supplemented with protease inhibitor (Sigma-Aldrich) for 30 min on ice then centrifuged at 14,000 rpm for 15 min at 4 °C. All lysates were prepared at a concentration of 1 × 10^6^ cells per 10 µL volume of lysis buffer. Whole cell lysates were boiled for 10 min and then resolved on a 12% Bis–Tris pre-cast gel (Thermofisher). Proteins were transferred onto a polyvinylidene difluoride membrane (Millipore) and then incubated in blocking buffer (LI-COR) for 30 min at room temperature. Primary antibody incubation using rabbit-anti-ZFP36 (Cell Signaling Technologies) and mouse-anti-β-actin (Invitrogen).

### Flow cytometry antibodies

The following antibodies were used (from BioLegend): CD3-BV711 (clone OKT3), OX40-FITC (clone ATC35); (from BD): Streptavidin-PE, CD4-PE-Cy5 (clone RPA-T4), CD8-APC-Cy7 (clone SK1), ICOS-BV650 (clone DX29), CD45RO-BV786 (clone UCHL1), CD62L-PE-Cy7 (clone DREG56), CD25-PE (clone M-A251); (from Jackson Laboratories): Biotin-SP-AffiniPure F(ab’)^2^ Fragment Goat Anti-Human IgG; (from Invitrogen): CD69-PerCP-eF710 (clone FN50).

### Mice

Animal experiments were performed according to experimental protocols approved by the Institutional Animal Care and Use Committee (IACUC) of the University of Pennsylvania. All methods were carried out according to IACUC guidelines. All methods performed on animals were reported in accordance to ARRIVE guidelines. Six- to eight-week-old male NOD/scid/IL2rg−/− (NSG) mice were procured from Jackson Laboratories and bred in the vivarium at the University of Pennsylvania and maintained in pathogen-free conditions.

### T cell enumeration

For peripheral blood human T cell counts, submandibular bleeds were performed to collect 50uL of blood for subsequent staining and lysis in Trucount™ tubes (BD).

### Cytotoxicity assay

Cytotoxic killing of target cells was assessed using a luminescence-based assay. T cells were co-cultured with luciferase expressing target cells at a range of effector:target (E:T) ratios in flat-bottom 96-well plates for 24 h at 37 °C in R10 media. Target cells were K562-Meso for mesoCAR-T cells. After 24 h, living target cells were quantified by the addition of luciferin substrate (GoldBio), and luciferase activity was measured after 10 min using the SpectraMax M3 plate reader (Molecular Devices). The percentage of lysis was determined using the following formula:

$${1}{-}\left[ {{\text{Signal}}_{{{\text{sample}}}} {-}{\text{Signal}}_{{\text{media only}}} \left] / \right[{\text{Signal}}_{{\text{tumor only}}} {-}{\text{Signal}}_{{\text{media only}}} } \right].$$

### Activation assay

A total of 1 × 10^5^ mesoCAR-T cells were co-cultured with K562-Meso overnight in 96-well round-bottom plates in 200 µL R10 media in triplicate. The next day, cells were prepared for flow cytometry staining of CD25 and CD69.

### Cytokine production

Supernatants were sampled from the activation assay (see above) for Luminex multiplex cytokine quantification (MilliporeSigma).

### Proliferation assay

A total of 5 × 10^4^ mesoCAR-T cells were stained with CellTraceViolet dye (Invitrogen) according to the manufacturer’s instructions and co-cultured with AsPC1 respectively at an E:T ratio of 1:3 in 96-well flat-bottom plates in 200 μL R10 media in triplicate for 1 week. Wells were topped off with R10 media at the midpoint of the assay to account for volume loss due to evaporation. After 1 week, cells were assayed via flow cytometry.

### Bulk RNA-seq

Total RNA was collected from 2 × 10^6^ T cells with the RNEasy Mini isolation kit (Qiagen). Library preparation and RNA-seq was performed by Novogene on the Illumina NovaSeq 6000 with paired-end 150-bp reads.

### RNA-seq data processing and analysis

Raw fastq files (.fq) were processed using Salmon alignment into counts files (.sf) using the LatchBio cloud-based bioinformatics platform (https://latch.bio/). Downstream analysis of counts files was performed using custom scripts in RStudio adapted from previously published scripts on GitHub (https://github.com/davemai/reg1-roq1-knockout).

### Supplementary Information

Supplementary Figure S1.

## Data Availability

RNA-seq data will be deposited onto Gene Expression Omnibus (GEO) accession number GSE234866. Code used to process RNA-seq data and generate plots is adapted from code previously published on GitHub (https://github.com/davemai/reg1-roq1-knockout). All other study data are included in the article and/or SI Appendix.
