# Supplementary material for: ZFP36 disruption is insufficient to enhance the function of mesothelin-targeting human CAR-T cells
Source: Sci Rep. 2024 Feb 7;14:3113. doi: 10.1038/s41598-024-53769-8 (PMC10850500; doi:10.1038/s41598-024-53769-8)
Supplement: Supplementary file 1 — Supplementary Figure S1. [file 41598_2024_53769_MOESM1_ESM.docx]

Supplementary information for “ZFP36 disruption is insufficient to enhance the function of mesothelin-targeting human CAR-T cells”

David Mai^1,2*^, Tifara Boyce^1¶^, Aakash Mehta^3,4¶^, Jordan Reff^2^, John Scholler^2^, Neil C. Sheppard^2,5^, Carl H. June^2,5^

*Corresponding author

Email: [damai@seas.upenn.edu](mailto:damai@seas.upenn.edu) (DM)

^¶^These authors contributed equally to this work.


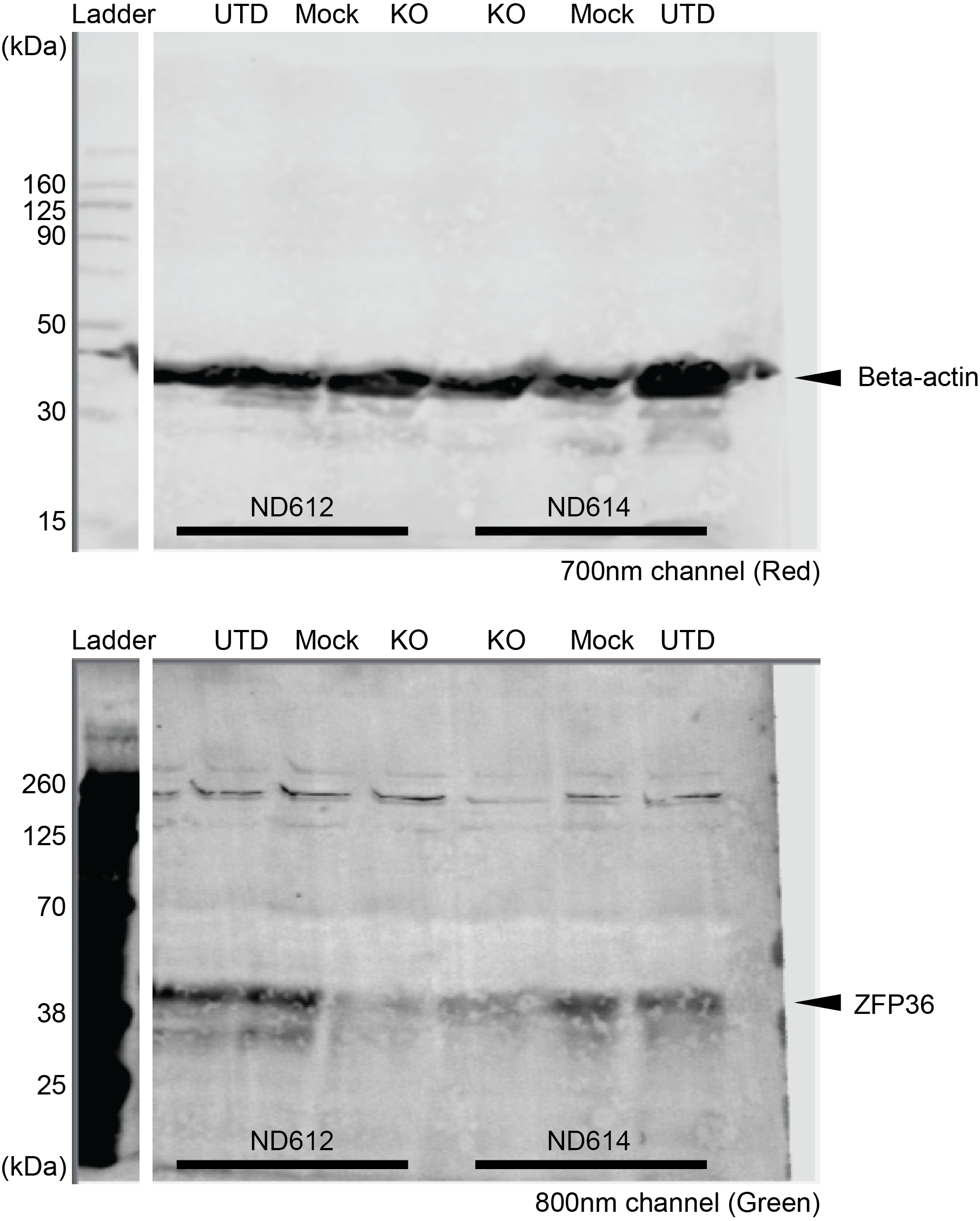


**Fig S1. Uncropped western blots from Fig 2C**. Full western blot images taken under the 700nm red channel (top) and 800nm green channel (bottom) to detect ZFP36 and β-actin as a loading control in human CAR-T cells.
